# P-1487. Respiratory syncytial virus (RSV) vaccination in adults aged <60 years at increased risk of severe RSV disease: considerations of the U.S. Centers for Disease Control and Prevention Advisory Committee on Immunization Practices

**DOI:** 10.1093/ofid/ofaf695.1671

**Published:** 2026-01-11

**Authors:** Michael Melgar, Amadea Britton, Diya Surie, Monica Godfrey, Ruth Link-Gelles, Fiona P Havers, Meredith L McMorrow, Mini Kamboj, Helen Y Chu, Keipp Talbot, Albert Shaw

**Affiliations:** Centers for Disease Control and Prevention, Atlanta, Georgia; Centers for Disease Control and Prevention, Atlanta, GA; Centers for Disease Control and Prevention, Atlanta, Georgia; Centers for Disease Control and Prevention, Atlanta, Georgia; Centers for Disease Control and Prevention, Atlanta, Georgia; Centers for Disease Control and Prevention, Atlanta, Georgia; CDC/NCIRD/CORVD/SPB, Atlanta, GA; MSKCC, New York, NY; University of Washington, Seattle, WA; Vanderbilt University Medical Center, Nashville, Tennessee; Yale School of Medicine, New Haven, Connecticut

## Abstract

**Background:**

Since June 2024, CDC’s Advisory Committee on Immunization Practices (ACIP) has recommended a single dose of RSV vaccination for all adults aged ≥75 years and for adults aged 60–74 years who are at increased risk of severe RSV disease. RSV vaccines are effective in preventing RSV-associated hospitalization, with the potential to prevent tens of thousands of annual hospitalizations and deaths in older adults. However, certain adults aged < 60 years are also at increased risk of severe illness caused by RSV.

**Methods:**

In considering RSV vaccination for adults aged 50–59 years, ACIP reviewed risk of RSV-associated hospitalization among adults with and without chronic medical conditions, differential risk by race and ethnicity, duration of vaccine protection and potential need for revaccination, risk of Guillain-Barre syndrome (GBS) associated with subunit RSV vaccines, national vaccine uptake, societal costs of the vaccination program, complexity of changes to the immunization schedule, and challenges in implementing a risk-based recommendation in retail pharmacies, where most older adults have received RSV vaccination.

**Results:**

ACIP members discussed variability in individual-level risk of severe RSV disease in persons with specific chronic conditions and the balance of benefits and risks of RSV vaccination in adults aged < 60 years, who generally experience lower risk of severe outcomes from RSV infection compared with older adults. Members were concerned about the disproportionate burden of severe RSV disease and higher prevalence of chronic medical conditions among Black adults in the U.S., particularly in the 50–59-year age group. Members expressed a need for more data to inform optimal timing of revaccination after vaccine-induced immunity wanes, which is particularly important for younger adults with longer remaining life expectancy.

**Conclusion:**

Overall, Committee members thought that the public health benefits of RSV vaccination outweighed risks for adults aged 50–59 years with certain chronic medical conditions. On April 16, 2025, ACIP recommended a single dose of RSV vaccine for these adults using the same qualifying risk conditions used for adults aged 60–74 years. ACIP plans to consider RSV vaccine recommendations for adults aged 18–49 years at a future public meeting.

**Disclosures:**

Helen Y. Chu, MD, MPH, Roche: Advisor/Consultant|Vir: Advisor/Consultant

